# Cardiac Troponin I: A Valuable Biomarker Indicating the Cardiac Involvement in Fabry Disease

**DOI:** 10.1371/journal.pone.0157640

**Published:** 2016-06-20

**Authors:** Christian Tanislav, Dursun Guenduez, Christoph Liebetrau, Anne Kathrin Giese, Sabrina Eichler, Nicole Sieweke, Maria Speth, Timm Bauer, Christian Hamm, Arndt Rolfs

**Affiliations:** 1 Department of Neurology, Justus Liebig University, Giessen, Germany; 2 Department of Cardiology and Angiology, Justus Liebig University, Giessen, Germany; 3 Kerckhoff Heart and Thorax Center, Department of Cardiology, Bad Nauheim, Germany; 4 German Centre for Cardiovascular Research (DZHK), partner site RheinMain, Frankfurt am Main, Germany; 5 Albrecht-Kossel Institute for Neuroregeneration, University of Rostock, Rostock, Germany; 6 Centogene, Rostock, Germany; 7 Department of Clinical Chemistry, Justus Liebig University, Giessen, Germany; KRH Robert Koch Klinikum Gehrden, GERMANY

## Abstract

**Objectives:**

Assessment of the clinical severity of Fabry disease (FD), an X-linked, rare, progressive disorder based on a genetic defect in alpha-galactosidase is challenging, especially regarding cardiac involvement. The aim of the study was to evaluate the diagnostic value of cardiac troponin I (cTnI) in discriminating FD patients with cardiac involvement in a large FD patient cohort.

**Methods:**

cTnI levels were measured with a contemporary sensitive assay in plasma samples taken routinely from FD patients. The assay was calibrated to measure cTnI levels ≥0.01 ng/ml. Elevated cTnI values (cut-off ≥0.04 ng/ml) were correlated with clinical data.

**Results:**

cTnI was assessed in 62 FD patients (median age: 47 years, males: 36%). Elevated cTnI levels were detected in 23 (37%) patients. Patients with a cTnI elevation were older (median 55 years versus 36 years, p<0.001). Elevated cTnI levels were associated with the presence of a LVH (16/23 versus 1/39; OR 65.81, CI: 6.747–641.859; p<0.001). In almost all patients with a left ventricular hypertrophy (LVH) elevated cTnI levels were detected (16/17, 94%). Absolute cTnI levels in patients with LVH were higher than in those without (median 0.23 ng/ml versus 0.02 ng/ml; p<0.001). A cTnI level <0.04ng/ml had a high negative predictive value regarding the presence of a LVH (38/39, 97%). In a control group of non-FD patients (n = 17) with LVH (due to hypertension) none showed cTnI levels ≥0.01 ng/ml.

**Conclusions:**

Elevated cTnI levels are common in FD patients, reflecting cardiac involvement. FD patients might benefit from a continuous cTnI monitoring.

## Introduction

Fabry disease (FD) is a rare, X-linked lysosomal storage disorder affecting hemizygous males and heterozygous females [1–4]. The intracellular storage affects different organ systems, causing severe alteration. Left ventricular hypertrophy indicates cardiac involvement in FD and is associated with a poor long-term outcome [5]. Since FD can be treated effectively, biomarkers for indicating the disorder and for monitoring the course of the disease are important [6–8]. Cardiac troponins I and T (cTnI and cTnT) are known to reflect acute and chronic myocardial injury. The introduced more sensitive cardiac troponin (cTn) assays now used in daily clinical practice have expanded the potential uses of troponin monitoring from risk stratification of patients with suspected acute coronary syndromes and diagnosis of myocardial infarction to mortality prediction of assumedly stable patients with coronary artery disease [9–11]. With this higher sensitivity the detection of minor myocardial injuries and/or myocardial stress is possible in patients with cardiac diseases other than coronary artery disease or acute myocardial infarction [12–14]. Approximately 60% of FD patients suffer from cardiac involvement, and few case series have demonstrated that cTnI levels are elevated in these patients [15–17]. Due to accumulation of sphingolipids in myocytes a continuous damage might lead to permanent elevation of troponins, which occurs without clinical correlates as known in acute myocardial ischemia [15,16]. The aim of the present study was to evaluate the diagnostic value of cTnI using a contemporary sensitive troponin I assay in order to discriminate FD patients with cardiac involvement in a large FD patient cohort.

## Methods

### Study settings and patients

Plasma samples taken routinely from patients with FD and stored at the Albrecht-Kossel Institute for Neuroregeneration, University of Rostock, Germany, were analyzed. All patients signed informed consent, which included consent for genetic and biomarker analyses. The ethics board of the University of Rostock, Germany, approved the study (A 2011 92). In all patients the diagnosis of FD was based on a molecular genetic analysis demonstrating a heterozygous or hemizygous mutation in the α-GAL-A-gene [18]. Baseline data and specific findings and symptoms relevant for the interpretation were recorded systematically in a database and were used for further analyses: medical history indicating the presence of angiokeratoma, acroparaesthesia, pain episodes, hypohydrosis, gastrointestinal symptoms, cornea verticillata, proteinuria, previous stroke, cerebral white matter lesions on brain MRI, extended diameter of the basilar artery, previous myocardial infarction, angina pectoris, left ventricular hypertrophy (LVH; defined as an interventricular septal and/or posterior wall thickness in end-diastole ≥13 mm as detected by echocardiography or cardiac MRI), arrhythmias (including paroxysmal or permanent atrial fibrillation and/or ventricular/supraventricular tachycardia and/or atrioventricular conduction delay II and III degree), hypertension, diabetes mellitus, hypercholesterolemia, and dialysis [19]. Hemodialysis may change the concentration of cardiac troponins [20]. FD patients requiring dialysis (n = 12) were therefore excluded from further analysis.

Age- and sex-matched individuals with LVH due to causes other than FD were included as a control group. The inclusion criteria for the control group were no FD mutation and LVH due to hypertension. The criteria for establishing the diagnosis of LVH in the control group were similar to FD patients. cTnI levels were compared between control individuals with LVH and FD patients. All individuals in the control group provided written informed consent for their participation in the study, and approval of the local ethics board of the University of Giessen was obtained. The investigation conforms to the principles outlined in the Declaration of Helsinki.

### Laboratory assessment

Venous blood samples were collected in EDTA-filled and gel-filled tubes during routinely performed clinical testing. Plasma and serum were processed immediately and frozen at −80°C at the Albrecht-Kossel Institute for Neuroregeneration, University of Rostock, Germany until assay. cTnI measurements were performed at the department of clinical chemistry of the Justus Liebig University Giessen, Germany. cTnI in plasma was measured with a sensitive immunoassay (ADVIA Centaur^®^ TnI-Ultra^™^ immunoassay, Siemens Medical Solutions Diagnostics, Tarrytown, NY, USA). The imprecision of the assay as reported in the package insert is characterized with a variation coefficient of 10% at a concentration of 0.03 ng/ml; for concentrations ≥0.04 ng/ml the variation coefficient is ≤10%. The recommended clinical decision limit for rule-out of acute myocardial infarction using this assay is <0.04 ng/ml, which represents the 99^th^ percentile of healthy volunteers. The lower detection limit for this cTnI assay is 0.006 ng/ml. In our study the test was calibrated to measure values ≥0.01 ng/ml. The imprecision at lower levels is higher with a variation coefficient >20% for values <0.01 ng/ml.

We defined cTnI levels <0.01 ng/ml as subnormal, normal cTnI levels as ≥0.01 ng/ml and <0.04 ng/ml, and elevated cTnI levels in case of measurements ≥0.04 ng/ml. The ADVIA Centaur^®^ TnI-Ultra^™^ immunoassay therefore fulfills the criteria mandated by the European Society of Cardiology and the American College of Cardiology of a highly sensitive test for discriminating myocardial infarction.

Lyso-globotriaosylceramide was measured in serum (lyso-Gb_3_, values >0.5 ng/ml were interpreted as elevated); measurements were performed at the Centogene AG, Rostock, Germany. Serum creatinine level was determined using the creatinin_2-kit for ADVIA Chemistry Systems (Bayer HealthCare, Tarrytown, NY, USA). The estimated glomerular filtration rate (eGFR) was calculated accordingly using the simplified MDRD equation: eGFR (ml/min/1.73m^2^ body surface) = 186 x (serum creatinine)^-1.154^ x (age)^-0.203^ x 0.742 (if female) x 1.212 (if black) [21].

### Statistical analysis

All data for continuous variables are expressed as median and interquartile range. Categorical variables are reported as frequencies and percentages. A normal distribution was verified by the Kolmogorov-Smirnov’s one-sample test. Nonparametric data was analyzed by applying the Mann–Whitney U-test. For comparing relative frequencies, the Fisher’s exact test was used. Relevant parameters for the interpretation and association with elevated cTnI levels in the univariate analysis were entered into a stepwise logistic regression analysis based on a forward likelihood procedure. For statistical analysis the SPSS (Statistical Package for Social Sciences) software, (version 22.0, SPSS Inc., Chicago, IL, USA) was used.

## Results

Out of 76 FD patients 62 were included in the analysis (excluded were 12 patients with dialysis and 2 further patients due to low quality of the blood samples). In the remaining 62 patients (median age 47.16 years) 36% were male. In 23 patients (37%) elevated cTnI levels (≥0.04 ng/ml) were detected (median 0.14 ng/ml [IQR 0.06–0.25 ng/ml]).

The clinical characteristics of all FD patients included in the study are shown in Table 1. Patients with elevated cTnI (≥0.04 ng/ml) were older (p<0.001) and had more often LVH (p<0.001), a history of myocardial infarction (p = 0.016), angina pectoris (p = 0.003) or arrhythmias (p = 0.03). Furthermore, patients with elevated cTnI had more often proteinuria (p = 0.016), lower eGFR values (p = 0.015) and higher lyso-Gb3 levels (p = 0.01).

**Table 1:** Patients with Fabry disease: comparison of patients with cTnI elevation ≥0.04 ng/ml versus without.

	Total cohort (n = 62)	cTnI elevation (n = 23)	No cTnI elevation (n = 39)	*P*-value
		(cTnI ≥0.04ng/ml)	(cTnI <0.04ng/ml)	
**Baseline data**				
Age, years; median (IQR*)	47.16 (31.88–56.56)	55.55 (47.27–61.08)	36.16 (24.41–49.60)	<0.001
Sex, male	22 (35.5%)	8 (34.8%)	14 (35.9%)	>0.999
**Fabry disease manifestation**				
Angioceratoma	21 (33.9%)	12 (52.2%)	9 (23.1%)	0.027
Acroparaesthesia	23 (37.1%)	9 (39.1%)	14 (35.9%)	>0.999
Pain episodes	32 (51.6%)	13 (56.5%)	19 (48.7%)	0.606
Hypohydrosis	21 (33.9%)	11 (47.8%)	10 (25.6%)	0.098
Gastrointestinal symptoms	17 (27.4%)	6 (26.1%)	11 (28.2%)	>0.999
Cornea verticillata	30 (48.4%)	14 (60.9%)	16 (41.0%)	0.189
Proteinuria	27 (43.5%)	15 (65.2%)	12 (30.8%)	0.016
Previous stroke	22 (35.5%)	10 (43.5%)	12 (30.8%)	0.411
White matter lesions, cerebral MRI	32 (51.6%)	16 (69.6%)	16 (41.0%)	0.038
Extended diameter of the basilar artery	22 (35.5%)	13 (56.5%)	9 (23.1%)	0.013
**Cardiovascular medical history**				
Previous myocardial infarction	4 (6.5%)	4 (17.4%)	0 (0%)	0.016
Angina pectoris	8 (13.1%)	7 (30.4%)	1 (2.6%)	0.003
Left ventricular hypertrophy	17 (27.4%)	16 (69.6%)	1 (2.6%)	<0.001
Arrhythmias	10 (16.1%)	7 (30.4%)	3 (7.7%)	0.030
Atrial fibrillation	7 (11.3%)	5 (21.7%)	2 (5.1%)	0.090
Hypertension	19 (30.6%)	10 (43.5%)	9 (23.1%)	0.153
Diabetes mellitus	5 (8.1%)	3 (13.0%)	2 (5.1%)	0.350
Hypercholesterolemia	5 (8.1%)	3 (13.0%)	2 (5.1%)	0.350
**Clinical chemistry**				
cTnI, ng/ml; median (IQR)	0.07 (0.02–0.23)	0.14 (0.06–0.25)	–	
Serum lyso-Gb3 (ng/ml); median (IQR)	5.43 (1.41–15.3)	7.17 (4.79–16.50)	2.93 (0.19–9.33)	0.010
Serum creatinine (mg/ml); median (IQR)	0.8 (0.6–0.93)	0.8 (0.7–1.2)	0.8 (0.6–0.9)	0.065
eGFR (ml/min/1.73 m^2^); median (IQR)	81.59 (67.35–108.35)	68.67 (59.45–93.02)	87.58 (69.01–122.17)	0.015

*Abbreviations: cTnI, cardiac troponin I; eGFR, estimated glomerular filtration rate; IQR, interquartile range; MRI, magnetic resonance imaging

For identifying factors independently associated with cTnI elevation, factors associated in the univariate analysis were entered into a stepwise logistic regression analysis. In a first step of the logistic regression analysis we tested a strong association between the factor age and the presence of LVH (LVH+ median 53.67 years [IQR 46.97–60.34 years] versus LVH- median 37.4 years [IQR 24.85–53.29 years]; p = 0.009); older patients might have a more advanced stage of the disease explaining higher rates of LVH. Therefore and, as the parameter LVH is a specific finding in FD and also potentially involved in a cTnI elevation, it was considered for the logistic analysis. The following parameters were included for calculating factors in the equation: LVH, lyso-Gb_3_, eGFR, and the presence of white matter hyperintensities in the cerebral MRI. The LVH remained the single factor independently associated with cTnI elevation (Table 2). The odds ratio for elevated cTnI for LVH was 65.8. For elevated cTnI levels (cut-off of ≥0.04 ng/ml) and the presence of a LVH we calculated a sensitivity of 94.1% (16/17), a specificity of 84.4% (38/45), a positive predictive value of 69.6% (16/23), and a negative predictive value of 97.4% (38/39).

**Table 2:** Multiple logistic regression analysis for elevated cardiac troponin (cut-off ≥0.04 ng/ml) patients with Fabry disease (n = 62).

	OR (95%CI)	*P*
eGFR*	0.986 (0.961–1.013)	0.309
Serum lyso-Gb3 level	0.996 (0.945–1.049)	0.869
Left ventricular hypertrophy	65.806 (6.747–641.859)	<0.001
White matter lesions, cerebral MRI	1.165 (0.223–6.071)	0.856

*Abbreviations: CI, confidence interval; eGFR, estimated glomerular filtration rate; MRI, magnetic resonance imaging; OR, odds ratio

In 17 patients with LVH, 16 had a cTnI elevation (≥0.04 ng/ml), one patient had a subnormal level (<0.01ng/ml). In 45 FD patients without a LVH elevated cTnI values (≥0.04 ng/ml) were found in 7 patients (7/45; median 0.06 ng/ml; range 0.04–0.14 ng/ml). Normal cTnI levels (≥0.01 ng/ml and <0.04 ng/ml) were detected exclusively in patients without a LVH (n = 11; median 0.01 ng/ml, [IQR 0.01–0.02 ng/ml]). In 28 patients subnormal cTnI values were found. Baseline characteristics in each cTnI category are presented in Table 3. Patients with cTnI elevation were older than those with a normal cTnI levels (median: 55.55 years [IQR: 47.27–61.08 years] versus median 32.67 years [IQR 21.47–49.78 years]; p = 0.004) (Table 3).

**Table 3:** Patients with Fabry disease dichotomized according to different categories of cTnI levels.

	cTnI elevation (n = 23)	Normal cTnI (n = 11)	Subnormal cTnI (n = 28)	*cTnI elevation versus normal cTnI*	*Normal cTnI versus subnormal cTnI*
	(cTnI ≥0.04ng/ml)	(<0.04 ng/ml and ≥0.01ng/ml)	(cTnI <0.01ng/ml)	*P*-value	*P*-value
**Baseline data**					
Age, years; median (IQR*)	55.55 (47.27–61.08)	39.40 (34.25–49.07)	32.67 (21.47–49.78)	0.004	0.593
Sex, male	8 (34.8%)	5 (45.5%)	9 (32.1%)	0.709	0.478
**Fabry disease manifestation**					
Angioceratoma	12 (52.2%)	4 (36.4%)	5 (17.9%)	0.477	0.238
Acroparaesthesia	9 (39.1%)	2 (18.2%)	12 (42.9%)	0.271	0.301
Pain episodes	13 (56.5%)	7 (63.6%)	12 (42.9%)	>0.999	>0.999
Hypohydrosis	11 (47.8%)	5 (45.5%)	5 (17.9%)	>0.999	0.109
Gastrointestinal symptoms	6 (26.1%)	3 (27.3%)	8 (28.6%)	>0.999	>0.999
Cornea verticillata	14 (60.9%)	4 (36.4%)	12 (42.9%)	0.274	>0.999
Proteinuria	15 (65.2%)	5 (45.5%)	7 (25.0%)	0.458	0.262
Previous stroke	10 (43.5%)	6 (54.5%)	6 (21.4%)	0.717	0.062
White matter lesions, cerebral MRI	16 (69.6%)	5 (45.5%)	11 (39.3%)	0.262	0.734
Extended diameter of the basilar artery	13 (56.5%)	4 (36.4%)	5 (17.9%)	0.465	0.238
**Cardiovascular medical history**					
Previous myocardial infarction	4 (17.4%)	0 (0%)	0 (0%)	0.280	>0.999
Angina pectoris	7 (30.4%)	0 (0%)	1 (3.6%)	0.057	>0.999
Left ventricular hypertrophy	16 (69.6%)	0 (0%)	1 (3.6%)	<0.001	>0.999
Arrhythmias	7 (30.4%)	0 (0%)	3 (10.7%)	0.069	0.545
Atrial fibrillation	5 (21.7%)	0 (0%)	2 (7.1%)	0.150	>0.999
Hypertension	10 (43.5%)	6 (54.5%)	3 (10.7%)	0.717	0.015
Diabetes mellitus	3 (13.0%)	2 (18.2%)	0 (0%)	>0.999	0.074
Hypercholesterolemia	3 (13.0%)	2 (18.2%)	0 (0%)	>0.999	0.074
**Clinical chemistry**					
cTnI, ng/ml; median (IQR)	0.14 (0.06–0.25)	0.01 (0.01–0.02)	–	–	
Serum lyso-Gb3 (ng/ml); median (IQR)	7.17 (4.79–16.50)	5.65 (3.03–27.70)	1.52 (0–7.8)	0.612	0.702
Serum creatinine (mg/ml); median (IQR)	0.8 (0.7–1.2)	0.7 (0.6–0.8)	0.8 (0.6–0.9)	0.133	0.990
eGFR (ml/min/1.73 m^2^); median (IQR)	68.67 (59.45–93.02)	92.30 (79.91–107.68)	86.92 (68.9–130.72)	0.071	0.406

*Abbreviations: cTnI, cardiac troponin I; eGFR, estimated glomerular filtration rate; IQR, interquartile range; MRI, magnetic resonance imaging

**Fig 1:**
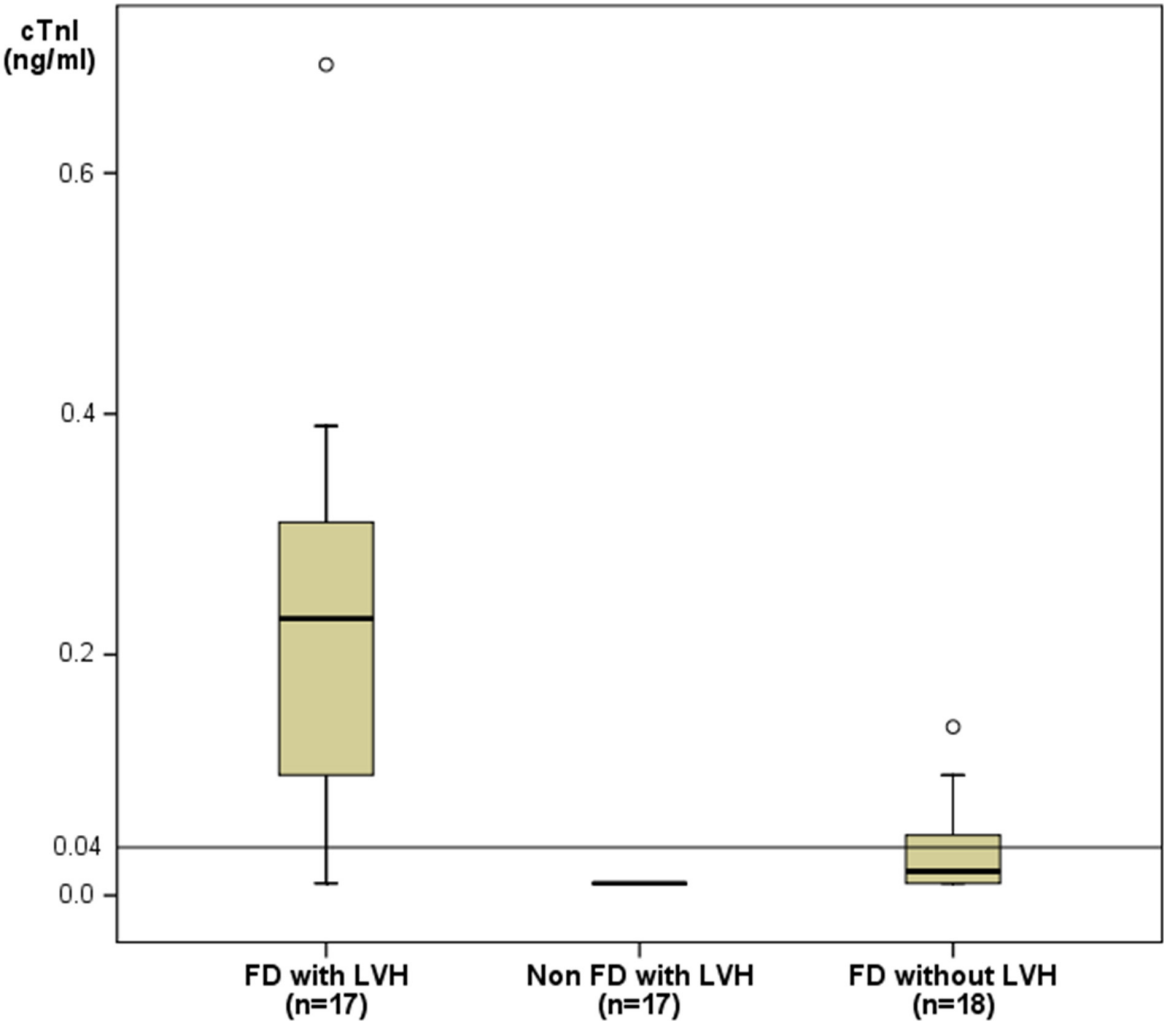
Comparison of cardiac troponin I levels. Presented are patients with Fabry disease (FD) and left ventricular hypertrophy (LVH) (n = 17) versus FD patients without LVH (n = 18) and non-FD patients with LVH of other cause (n = 17). The cut-off level for cTnI in diagnosing myocardial infarction (≥0.04 ng/ml) is indicated in the figure.

FD patients with LVH had more often elevated or subnormal cTnI levels compared with FD patients without LVH (16/17 versus 18/45; p = 0.002). Accordingly, absolute cTnI levels in FD patients with LVH were higher than in FD patients without LVH (0.23 ng/ml [IQR 0.09–0.32 ng/ml] versus 0.02 ng/ml [IQR 0.01–0.05 ng/ml]; p<0.001). In the matched control group of non-Fabry subjects (n = 17, baseline data are summarized in Table 4) cTnI (≥0.01 ng/ml) was detected in none of the participants (non FD: 0/17 versus FD: 16/17, p<0.001), (Fig 1).

**Table 4:** Characteristics in 17 age- and sex-matched individuals with left ventricular hypertrophy due to hypertension.

	FD patients with LVH (n = 17)	Non-FD participants with LVH (n = 17)	*P*-value
**Baseline data**			
Age (years); median (IQR)	53.67 (46.97–60.30)	53.23 (46.15–59.81)	>0.999
Sex (male)	7 (41.2%)	7 (41.2%)	>0.999
Hypertension	8 (47.1%)	17 (100%)	<0.001
Diabetes mellitus	2 (11.8%)	0 (13.0%)	0.145
Hypercholesterolemia	2 (11.8%)	0 (13.0%)	0.145
**Clinical chemistry**			
cTnI, ng/ml median (IQR)	0.23 (0.09–0.32)	–	
Serum creatinine, mg/ml; median (IQR)	0.8 (0.7–1.2)	0.9 (0.7–1.3)	>0.999
eGFR, ml/min/1.73 m^2^; median (IQR)	75.11 (56.08–92.62)	71.28 (54.25–90.12)	>0.999

Abbreviations: cTnI, cardiac troponin I; eGFR, estimated glomerular filtration rate; FD, Fabry disease; IQR, interquartile range; LVH, left ventricular hypertrophy.

## Discussion

Cardiac involvement in FD is mainly related to progressive cardiac hypertrophy and is linked to a poor prognosis [22–24]. Cardiomyocytes of FD patients are affected by storage of Gb_3_, which leads to structural damage of the myocardium [23,25]. Therefore, timely and adequate risk stratification in FD patients is essential.

Previous investigations found elevated cTn levels in FD patients with LVH, consistent with the presence of myocardial injury [15–17]. In contrast to his exceptional relevance in the diagnosis of an acute myocardial ischemia, troponins in FD seams to reflect the continuous damage in myocytes due to accumulation of sphingolipids [15–17]. In this context, the present study is the first investigation of the diagnostic value of cTnI in FD using a contemporary sensitive TnI assay in order to discriminate between FD patients with and without cardiac involvement. The most important finding of our study is that LVH as a possible surrogate for cardiac involvement in FD patients can be safely ruled out when cTnI measurements are in the normal or subnormal range; we calculated a high negative predictive value of 97.4% when cTnI <0.04 ng/ml. In our study an elevated cTnI level (≥0.04 ng/ml) was found in almost in every FD patient with LVH (94.1%). FD patients with elevated cTnI values had an OR of 65.6 for having LVH. Only one FD patient had a cTnI level in the subnormal range; this leads to the speculation that this patient had LVH of an etiology other than FD. This is in line with findings from the age- and sex-matched control group consisting of individuals with LVH mainly due to hypertension who did not have elevated cTnI.

A considerable proportion of patients in our cohort hat normal cTnI levels (≥0.01 ng/ml and <0.04 ng/ml) with no abnormal diagnostic finding regarding cardiac involvement. The presence of cTnI in these patients with no cardiac symptoms could be interpreted as a first indicator of cardiac involvement in FD, prior typical morphological and clinical findings occur. In this regard, determination of cTnI has both diagnostic and prognostic value as the specific enzyme replacement therapy for FD has the greatest impact if started at an early stage of the disease, reducing morphological and functional organ changes [26]. Lyso-Gb_3_ is a biomarker in FD that is closely related to the disease severity [27], and the association between cTnI elevation and an increase in lyso-Gb_3_ in our patients is also an indication of the value of cTnI in assessing disease progression. Thus, proving the prognostic value of troponins in FD (even levels below 0.01 ng/ml, detectable in highly sensitive tests) is of high clinical relevance and has implications for therapy decision making.

We observed that elevated cTnI levels correlated markedly with cardiac manifestation of FD. Based on our findings, cTnI would seem to be the ideal biomarker to incorporate into a diagnostic algorithm that utilizes these results, an algorithm that would facilitate an early decision for starting therapy. Thus, if the interval between the diagnosis of cardiac involvement and treatment could be shortened, the resulting earlier treatment period for such patients would have major resource implications. This validation would have to be performed in a large-scale, real-world scenario, however, to establish appropriate screening protocols for FD patients.

Several disease entities other than coronary artery disease have been described to be associated with elevated cardiac troponin levels, including critical illness, sepsis, stroke, or epileptic seizure [15,28–34]. Within this spectrum FD should be considered as a differential diagnosis if elevated cardiac troponins of unknown etiology are detected. In this context, renal disease has been described as a potential cause of cTn elevation [28,30,31]. Troponin has been known to be elevated in the setting of even mild cases of renal failure [35]; however, cTn is independently related to LV mass and predicts all-cause and cardiovascular mortality even in hemodialysis patients [36]. Furthermore, there is no difference in the half-life and the elemination rate constant of cTnI in patients with acute myocardial infarction and end-stage renal disease when compared with patients having normal kidney function [37]. Therefore, elevated cTn levels in FD patients with renal disease may be better explained due to cardiac involvement caused cardiac injury, especially when the levels are not changing rapidly over time. Regarding our FD patients with need for dialysis normal and elevated cTnI levels (≥0.01ng/ml) were more frequently observed. However, FD patients with end-stage renal disease seemed to be more affected by the disease, showing also more frequently typical FD findings.

### Limitations

This study provides evidence of the diagnostic value of cTnI in discriminating between FD patients with and without cardiac involvement. A limitation of our study that must be considered, however, is that it makes a retrospective analysis of prospectively collected data. Also the small sample size of investigated patients might be a further limitation of the presented study. However, considering Fabry disease is a rare pathology, with 62 participants we assembled a remarkable group of patients. The data were furthermore sufficient to demonstrate the value of cTnI in the diagnosis of cardiac involvement in patients with FD.

Other factors potentially causing an elevation of troponins need to be taken into consideration when interpreting our results. Nevertheless, regarding this issue we tended to avoid bias as far as possible. In the context of the cardiac pathology in Fabry disease our results, consisting of a high sensitivity for elevated cTnI in patients with LVH (16/17, 94.1%) and the high negative predictive value (38/39, 97.4%) for regular troponin levels in the absence of a LVH, appears plausibly and underlines the reliability.

## Conclusions

cTnI seems to be a valuable and valid surrogate marker for indicating cardiac involvement in FD, which can be safely ruled out when cTnI is below the 99^th^ percentile. Further investigations of the diagnostic and prognostic value of cardiac troponins in FD are warranted as knowledge about their levels in plasma might potentially represent a first cardiac-specific pathological finding in the course of the disease. This is especially relevant for an early decision about initiating enzyme replacement therapy.

## Abbreviations

cTn: cardiac troponin
cTnI: cardiac troponin I
cTnT: cardiac troponin T
FD: Fabry disease
eGFR: estimated glomerular filtration rate
α-GAL-A: α galactosidase A
Gb_3_: globotriaosylceramide
MRI: magnet resonance imaging
LVH: left ventricular hypertrophy
